# Cracking the Code of Oocyte Quality: The Oxidative Stress Link to IVF Success

**DOI:** 10.3390/ijms26136377

**Published:** 2025-07-02

**Authors:** Charalampos Voros, Diamantis Athanasiou, Ioannis Papapanagiotou, Despoina Mavrogianni, Antonia Varthaliti, Kyriakos Bananis, Antonia Athanasiou, Aikaterini Athanasiou, Georgios Papadimas, Athanasios Gkirgkinoudis, Kyriaki Migklis, Dimitrios Vaitsis, Aristotelis-Marios Koulakmanidis, Charalampos Tsimpoukelis, Sofia Ivanidou, Anahit J. Stepanyan, Maria Anastasia Daskalaki, Marianna Theodora, Panagiotis Antsaklis, Dimitrios Loutradis, Georgios Daskalakis

**Affiliations:** 1 1st Department of Obstetrics and Gynecology, ‘Alexandra’ General Hospital, National and Kapodistrian University of Athens, 80 Vasilissis Sofias Avenue, 11528 Athens, Greece; depy.mavrogianni@yahoo.com (D.M.); antonia.varthaliti@hotmail.com (A.V.); aristoteliskoulak@gmail.com (A.-M.K.); tsimpoukelischa@gmail.com (C.T.); md181341@students.euc.ac.cy (M.A.D.); martheodr@gmail.com (M.T.); panosant@gmail.com (P.A.); gdaskalakis@yahoo.com (G.D.); 2 IVF Athens Reproduction Center V. Athanasiou, 15123 Maroussi, Greece; diamathan16@gmail.com (D.A.); antoathan16@gmail.com (A.A.); diamathan17@gmail.com (A.A.); 3 Athens Medical School, National and Kapodistrian University of Athens, 15772 Athens, Greece; gpapamd@hotmail.com (I.P.); dr.georgepapadimas@gmail.com (G.P.); kyriaki.migklis@gmail.com (K.M.); vaitsisdim@gmail.com (D.V.); info@ivanidou.gr (S.I.); anahitstepanyanj@gmail.com (A.J.S.); loutradi@otenet.gr (D.L.); 4 King’s College Hospitals NHS Foundation Trust, London SE5 9RS, UK; kyriakos.bananis@nhs.net; 5 Fertility Institute-Assisted Reproduction Unit, Paster 15, 11528 Athens, Greece

**Keywords:** perfluoroalkyl substances, female infertility, ovarian function, in vitro fertilization (IVF), endocrine disruptors

## Abstract

The success of in vitro fertilization (IVF) and female reproductive capacity are significantly determined by oocyte quality. Increasing data highlights the significance of oxidative stress—a state of imbalance between reactive oxygen species (ROS) and antioxidant defenses—in regulating oocyte competence. Normal folliculogenesis and ovulation rely on optimal ROS levels; excessive oxidative stress (OS) can lead to DNA fragmentation, undermine meiotic spindle integrity, and trigger apoptosis in cumulus and granulosa cells. Molecular insults impair nuclear and cytoplasmic maturation, thereby impacting fertilization potential and embryonic development. Individuals with polycystic ovarian syndrome (PCOS), endometriosis, advanced maternal age, and metabolic disorders—conditions associated with suboptimal IVF outcomes—frequently exhibit redox imbalance. This narrative review examines significant oxidative markers in the follicular environment, exploring the molecular processes linking OS to diminished oocyte quality and discussing therapy techniques aimed at mitigating oxidative damage. Maintaining redox homeostasis in the ovarian milieu appears to be an effective strategy for enhancing oocyte competence and optimizing outcomes in assisted reproduction.

## 1. Introduction

The primary determinant of female reproductive potential is the developmental competency of a human oocyte, which enables it to initiate and complete meiosis, achieve successful fertilization, and sustain early embryonic development [1]. In the context of assisted reproductive technologies (ARTs), particularly IVF, the intrinsic quality of the oocyte significantly influences embryo viability, implantation rates, and the sustainability of pregnancy [2]. The quality of oocytes continues to pose a difficulty despite significant advancements in ovarian stimulation methods, embryonic culture systems, and cryopreservation procedures. In women with PCOS, endometriosis, unsolved infertility, or advanced maternal age, clinical failure in IVF is frequently due to diminished developmental potential of oocytes rather than their absence. Current clinical markers, such as anti-Müllerian hormone (AMH), follicle-stimulating hormone (FSH), and antral follicle count (AFC), just estimate ovarian reserve rather than assess oocyte quality [3,4,5].

OS, a disruption in the equilibrium between the production of ROS and the antioxidant defense mechanisms that maintain cellular redox homeostasis, is one of the most extensively studied molecular factors contributing to suboptimal oocyte quality [6]. While low levels of ROS, such as superoxide anion O_2_^−^, hydrogen peroxide H_2_O_2_, and hydroxyl radical ·OH, are essential for physiological functions like folliculogenesis, ovulation, and steroidogenesis, elevated concentrations of ROS initiate molecular damage within the ovarian microenvironment [7]. ROS disrupt intracellular signaling, induce oxidative modifications of DNA, lipids, and proteins, consequently initiating apoptosis and mitochondrial dysfunction [8,9]. The primary sources of ROS in the follicle encompass environmental or iatrogenic factors such as gonadotropin hyperstimulation, inflammation, or metabolic dysregulation; mitochondrial ETC leakage, particularly from complexes I and III; and NADPH oxidase (NOX) activity [10].

Oxidative damage to oocytes is facilitated at the intracellular level by numerous interconnected pathways. Mitochondria, which are maternally inherited and crucial for egg development, are especially susceptible to oxidative damage. ROS accumulation induces the opening of the mPTP, resulting in the depolarization of the mitochondrial membrane potential (ΔΨm) and the release of cytochrome c, thereby activating caspase-9 and caspase-3 in the intrinsic apoptotic pathway [11,12]. Essential processes for efficient meiosis II and cytoplasmic maturation—spindle assembly, chromosomal alignment, and organelle redistribution—are hindered by mitochondrial dysfunction.

Redox imbalance also disrupts nuclear processes. ROS stimulate the oxidation of guanine, resulting in double-stranded breaks, the activation of the DNA damage response (DDR), and the cessation of meiotic growth, characterized by the formation of 8-hydroxy-2′-deoxyguanosine (8-OHdG) [13]. ROS simultaneously target polyunsaturated fatty acids in membranes, initiating lipid peroxidation and generating reactive aldehydes such as MDA and 4-hydroxynonenal (4-HNE), which further destabilize the oolemma, impede sperm binding, and disrupt intracellular Ca^2+^ signaling essential for oocyte activation following fertilization [14]. Meiotic aneuploidy also arises from protein carbonylation, which disrupts cytoskeletal proteins essential for spindle stability.

Transcriptional and epigenetic control is also altered during OS. Redox-sensitive transcription factors including as NF-κB, AP-1, and Nrf2, which regulate inflammatory and antioxidant responses, are activated by ROS [15]. ROS interfere with the cAMP-PKA, PI3K-AKT, and MAPK signaling pathways in the oocyte–granulosa cell complex, hence altering gene expression associated with meiosis, apoptosis, and mitochondrial biogenesis [10]. OS significantly suppresses peroxisome proliferator-activated receptor gamma coactivator 1-alpha (PGC-1α), a major regulator of mitochondrial biogenesis, hence reducing mtDNA copy number and respiratory chain activity [9]. This induces a metabolic crisis in the egg, rendering it ineffective for fertilization and subsequent development.

Clinical research corroborates these mechanisms. Follicular fluid (FF) is enriched in ROS and NADPH oxidase isoforms, particularly NOX2, in patients with PCOS [16]. ROS accumulation in granulosa cells (GCs) leads to cell death via mitochondrial pathways and the downregulation of oocyte-supportive genes such as *GDF9* and *BMP15*. Pharmacological inhibition of NOX2 restores GC viability and reduces ROS production [3]. In endometriosis, FF exhibits elevated levels of nitric oxide (NO), iron, cadmium, and lead, alongside diminished antioxidant enzymes such as superoxide dismutase (SOD), glutathione peroxidase (GPx), catalase (CAT), and glutathione (GSH)—a milieu associated with decreased fertilization rates and impaired blastocyst development [4,17].

Recent findings indicate that ovarian tissues in women with endometriosis and metabolic inflammation exhibit activation of the NLRP3 inflammasome, a ROS-sensitive cytosolic complex [18]. The activation induces caspase-1 cleavage and the maturation of IL-1β and IL-18, thereby initiating pyroptosis in germ cells and exacerbating local inflammation, which disrupts follicular integrity and compromises oocyte maturation [19]. Advancing age exacerbates these redox imbalances further. Aged oocytes are characterized by diminished telomerase activity, mitochondrial DNA deletions, and modified expression of antioxidant enzymes and repair proteins [20]. The decline in SIRT1 and FOXO3a activity in aged oocytes compromises mitochondrial integrity and genomic stability, hence heightening susceptibility to ROS. Aneuploid embryos arise from compromised SAC mechanisms, resulting in chromosomal nondisjunction. These factors elucidate why oocyte quality, rather than oocyte quantity, emerges as the primary impediment for women over 35 undergoing IVF [12].

The evaluation of OS markers in FF—specifically, total oxidative status (TOS), oxidative stress index (OSI), 8-hydroxydeoxyguanosine (8-OHdG), malondialdehyde (MDA), and peroxiredoxin-4 (PRDX4)—has been proposed as a non-invasive approach for assessing oocyte viability. Low fertilization rates and suboptimal embryo morphology are associated in FF with diminished levels of TAC, GSH, and PRDX4 [21,22,23]. Furthermore, elevated IL-10 and diminished levels of IL-6, IL-8, and HIF-1α in FF are indicative of embryo viability and implantation potential [24]. This data highlights a distinct correlation between reproductive potential and the follicular redox environment.

This narrative review critically examines the molecular routes by which OS diminishes oocyte development and fertilization competence. We will investigate the primary oxidative mechanisms inside the follicular niche, identify predictive markers of oocyte quality, and assess therapeutic interventions—from lifestyle and dietary antioxidants to targeted pharmacological agents—aimed at altering redox equilibrium. Elucidating the redox biology of oocyte competence will facilitate the development of innovative treatment strategies to improve IVF outcomes and fertility preservation.

## 2. Physiological Role of ROS in Ovarian Folliculogenesis

Although typically associated with cytotoxicity and cellular damage, ROS play crucial roles as precisely controlled signaling molecules in ovarian physiology [25]. Low to moderate levels of ROS function as intracellular second messengers, facilitating various fundamental processes including metabolic adaptation, gene expression, kinase activation, and cytoskeletal remodeling [26]. The intricate processes of folliculogenesis and ovulation, which need precise temporal and spatial regulation of ROS levels to ensure follicle maturation and oocyte competence, are particularly reliant on this redox-sensitive signaling [27].

Initial follicular development ROS are intrinsically generated in GCs and theca cells by mitochondrial oxidative phosphorylation, several isoforms of NADPH oxidase (NOX1–4), and the action of xanthine oxidase [28]. This basal ROS production regulates critical signaling pathways such as phosphoinositide 3-kinase/protein kinase B (PI3K/AKT) and mitogen-activated protein kinase (MAPK/ERK) instead of causing harm [29]. These pathways regulate survival, follicular development, and germinal center proliferation. The ROS-mediated activation of PI3K/AKT influences the transcription of cyclin D2, an essential regulator of the GC cell cycle, and contributes to the stabilization of hypoxia-inducible factor 1-alpha (HIF-1α), hence promoting angiogenesis through vascular endothelial growth factor (VEGF). This vascular expansion ensures the growing follicle receives adequate oxygen and nutrient supply [30].

ROS simultaneously regulate steroidogenesis in GCs by altering the transcription of key enzymes such as steroidogenic acute regulatory protein (STAR), cytochrome P450 side-chain cleavage enzyme (CYP11A1), and 3β-hydroxysteroid dehydrogenase (HSD3B1) [31]. The activation of transcription factors, such as steroidogenic factor 1 (SF-1) and nuclear factor kappa B (NF-κB), triggered by ROS, regulates the generation of progesterone and estradiol, thereby mediating this control [32]. The maintenance of bidirectional communication between the oocyte and its surrounding somatic cells is contingent upon hormone action.

ROS levels temporarily increase in response to the LH surge during the preovulatory phase of follicle development. The upregulation of NOX2 and mitochondrial complex III activity in GCs and cumulus cells (CCs) elucidates the physiological rise in ROS. In conjunction with LH, the elevated ROS facilitate the activation of epidermal growth factor receptor (EGFR) signaling through transactivation mechanisms reliant on ROS-induced disulfide bond formation within EGFR domains [33]. Crucial for cumulus formation, oocyte maturation, and follicular rupture, this signaling cascade subsequently activates extracellular signal-regulated kinases (ERK1/2) and p38 MAPK.

ROS also influence cAMP-PKA signaling, which regulates meiotic arrest and resumption. Elevated intra-oocyte cAMP concentrations, maintained by G-protein-coupled receptor 3 (GPR3) activation and gap junctional communication with CCs, contribute to the preservation of meiotic arrest [34]. ROS facilitate the activation of phosphodiesterase 3A (PDE3A) during the LH surge, resulting in cAMP breakdown, an elevation in maturation-promoting factor (MPF) activity, and the progression of oocyte development from prophase I to metaphase II. This redox-sensitive checkpoint emphasizes the significant role of ROS in orchestrating somatic and germ cell development [35]. Furthermore, ROS, significant mediators of cumulative matrix remodeling, influence the expression of hyaluronan synthase 2 (HAS2) and prostaglandin-endoperoxide synthase 2 (PTGS2) in the COC. The release of oocytes post ovulation and their subsequent capture by the fallopian tube are contingent upon this rearrangement.

Beyond their impact on mitochondrial integrity and classical signaling pathways, ROS control a spectrum of post-transcriptional and translational regulating mechanisms vital throughout the late follicular phase [36]. Target one is the regulation of oocytes and granulosa cell (GC) RNA-binding proteins (RBPs). ROS have been found, for instance, to affect the activity of ELAVL1/HuR, an RBP that stabilizes transcripts encoding ovarian factors and antioxidant enzymes [37]. Redox-sensitive HuR translocation from the nucleus to the cytoplasm is necessary for post-LH surge production of cyclooxygenase-2 (COX-2), hyaluronan synthase 2 (HAS2), and tumor necrosis factor alpha-induced protein 6 (TNFAIP6), which help to contribute to cumulative matrix enlargement and oocyte release.

Concurrently, physiological ROS oscillations fine-tune the redox-sensitive kinome in cumulus–oocyte complexes (COCs). Among the kinases involved, LKB1–AMP-activated protein kinase (AMPK) senses intracellular ATP/ROS equilibrium [38]. Increased AMP levels and mild oxidative stimuli activate AMPK, which promotes mitochondrial biogenesis via activation of peroxisome proliferator-activated receptor gamma coactivator 1-alpha (PGC-1α) and reduces mTOR signaling, therefore preserving oocyte energy for meiotic progression [39]. During the metabolically difficult transition of oocytes from the germinal vesicle to metaphase II stage, such redox–AMPK coordination guarantees energy homeostasis.

ROS also regulate gap junction intercellular communication (GJIC), which is crucial for the interchange of ions, nucleotides, and metabolic intermediates between GCs and the oocyte, in the follicular niche [40]. Particularly, ROS control the phosphorylation state of connexin-43 (Cx43), the main gap junction protein in COCs. Moderate ROS levels can momentarily disturb Cx43 phosphorylation at serine 368, a change that closes gap junctions in response to LH and helps to enable the timely meiotic resuming [41]. But oocyte hydration and nutrient absorption, processes directly related to developmental competency, depend on the closely timed re-opening of these junctions.

Moreover, during late folliculogenesis redox-sensitive microRNA (miRNA) expression patterns are actively rebuilt. Recent research shows that ROS can cause particular miRNAs in GCs—such as miR-21 and miR-155—which reduce pro-apoptotic targets (e.g., PDCD4, FOXO1) and encourage luteinization to be expressed [42]. Exosomes leak these miRNAs into the FF and may also have paracrine effects on surrounding cells, therefore revealing an epigenetic layer of redox-mediated control [43].

Crucially, ROS are linked to control of oocyte spindle dynamics via oxidative changes of tubulin subunits and mitotic spindle-associated proteins. Transient ROS peaks coordinate actin nucleation by affecting cytoskeletal remodeling via Rho-family GTPases (e.g., RAC1, CDC42), therefore facilitating appropriate spindle migration and anchoring [44]. Redox management guarantees asymmetric spindle placement in this environment, which is necessary for both oocyte ploidy maintenance and effective extrusion of the first polar body.

Furthermore, important for ovulation is follicular immune privilege, which ROS helps to produce. ROS function as upstream controllers of pro-inflammatory mediators including prostaglandin E2 (PGE2), interleukin-8 (IL-8), and chemokine (C-C motif), by adjusting prostaglandin synthesis and leukocyte infiltration into the follicular wall [45]. These immunological signals encourage follicular wall breakdown, rupture, and corpus luteum development that follows.

Ultimately, the intra-follicular antioxidant system preserves cellular integrity by working in tandem with ROS formation. Under modest OS, the nuclear translocation of nuclear factor erythroid 2-related factor 2 (Nrf2) causes transcription of a battery of cytoprotective genes including heme oxygenase-1 (*HO-1*), NAD(P)H quinone dehydrogenase 1 (NQO1), and glutamate–cysteine ligase (GCL) [46]. These enzymes retain a redox environment appropriate for oocyte survival and meiotic integrity.

Moreover, recent data indicates that follicular autophagy, a mechanism crucial for maintaining cellular quality control and organelle turnover throughout follicle development, is predominantly ROS [47]. The basal autophagic flow in GCs enhances steroidogenic function and viability after temporary metabolic or oxidative disturbances. ROS regulate autophagy through redox-sensitive modulation of the AMPK–motor–ULK1 axis, wherein AMPK activation under moderate oxidative conditions inhibits motor complex 1 (motorC1) and initiates autophagosome formation via ULK1 phosphorylation [48]. This balance ensures the removal of oxidized proteins and faulty mitochondria (mitophagy), hence preserving cytoplasmic homeostasis in germ cells and indirectly supporting oocyte viability.

The impact of ROS on extracellular vesicle (EV) formation and signaling within the follicular environment is further underestimated. GCs and CCs secrete EVs, such as exosomes and microvesicles, which are enriched with miRNAs, long noncoding RNAs (lncRNAs), enzymes, and metabolic intermediates that influence oocyte maturation [49]. Recent experiments have demonstrated that transient increases in ROS enhance the expression of tetraspanins (e.g., CD9, CD81) and components of the endosomal sorting complex required for transport (ESCRT), hence facilitating exosomal release [50]. These redox-sensitive EVs regulate pathways associated with oocyte growth, epigenetic alteration, and cumulus enlargement, thereby serving as conduits for paracrine and juxtacrine communication between somatic and germline compartments.

Moreover, physiological ROS regulate the redox state of thiol-containing proteins, particularly protein disulfide isomerases (PDIs) and thioredoxins, which are essential for proper protein folding, zona pellucida hardening, and cortical granule formation [51]. These redox chaperones facilitate extracellular matrix synthesis and sperm-binding capability both within the egg and in adjacent CCs. Importantly, ROS influence the metabolism of metabolites such as glutamate, taurine, and cysteine, along with precursors for glutathione synthesis and osmolytes essential for oocyte hydration [52]. For instance, the control of cystine/glutamate transporters (xCT) in GCs by ROS directly affects intracellular glutathione availability and redox buffering capacity. This subsequently influences the mitochondrial redox equilibrium within the oocyte, hence emphasizing the link between somatic and germline compartments.

Cyclic redox oscillations have finally been proposed as temporal signals for meiotic development. Redox cycles may align with circadian gene expression networks through BMAL1–CLOCK complexes, thus integrating systemic endocrine signals with the local redox state, according to research in mammalian oocytes. These findings indicate a potential role of ROS in synchronizing oocyte competence with the reproductive axis and circadian control, particularly concerning ovarian age and infertility associated with shift work. These intricate redox-regulated systems collectively characterize ROS as diverse modulators of folliculogenesis, establishing a balance among structural integrity, metabolic activity, and signaling adaptability. The subsequent section will elucidate how extended OS disrupts this physiological mechanism and its implications for oocyte maturation and fertility.

We created a focused Table 1 summarizing relevant papers that elucidate the molecular mechanisms of ROS-mediated regulation during folliculogenesis and oocyte maturation to synthesize the physiological roles of ROS in ovarian function. The selected references ensure a translational perspective by integrating in vitro cellular models, animal studies, and human FF analysis. The papers were chosen based on their methodological rigor, relevance to the specific redox pathway under examination, and their contribution to understanding how ROS modulate the metabolic, epigenetic, and structural characteristics of the oocyte and its surrounding somatic cells. To provide a cohesive molecular framework that aligns with the comprehensive scope of our investigation, we prioritized quality over exhaustiveness, akin to a systematic review.

**Table 1:** Molecular mechanisms through which physiological ROS regulate folliculogenesis and oocyte maturation: selected evidence from the literature.

Mechanism	Molecular Players/Pathways	Target Cell Type	Oocyte-Specific Effect	Physiological Outcome	References
Controlled basal ROS signaling	Mitochondrial OXPHOS, NOX1-4, xanthine oxidase → PI3K/AKT, ERK1/2	Granulosa cells (GCs)	Indirect: supports GC proliferation → oocyte nourishment	Follicle growth, GC survival, early angiogenesis (via HIF-1α/VEGF)	Agarwal et al., 2005; Artini et al., 2022 [8,24]
LH-triggered ROS peak	NOX2, DUOX, mitochondrial burst → EGFR–ERK1/2–p38 MAPK	GCs, Cumulus cells (CCs)	Indirect: enhances cumulus expansion → meiotic resumption	Prepares cumulus–oocyte complex for ovulation	Wang et al., 2021 [9]
Mitochondrial remodeling	OPA1, MFN1/2, DRP1, ROS → actin cytoskeleton modulation	Oocyte	Ensures spindle asymmetry, correct chromosome alignment	Cytoplasmic maturation, prevention of aneuploidy	Sasaki et al., 2020 [12]
Redox-sensitive cAMP signaling	ROS → PDE3A activation → ↓ cAMP → ↑ MPF	Oocyte	Triggers meiosis I resumption, germinal vesicle breakdown	Nuclear maturation (MII stage entry)	Rodríguez-Varela & Labarta, 2020 [11]
Autophagy regulation	AMPK ↑ → mTORC1 ↓ → ULK1 ↑	GCs	Indirect: supports GC stress tolerance and steroidogenesis	Maintains GC viability under oxidative load	Wang et al., 2021; Moustakli et al., 2024 [9,19]
EV biogenesis under ROS control	CD63, CD9, CD81, ESCRT → exosome release	CCs, GCs	miRNA and protein delivery to oocyte via exosomes	Epigenetic modulation, chromatin remodeling	Lee et al., 2024 [53]
Thiol–redox protein remodeling	Thioredoxin, PDIs, ROS-sensitive cysteines	Oocyte and CCs	Protein folding, cortical granule formation, zona pellucida hardening	Fertilization competence, block to polyspermy	Vogelsang et al., 2024 [54]
Redox modulation of GJIC	Cx43 phosphorylation (Ser368)	CCs–Oocyte	Temporary closure → resumption of meiosis	Sync of cytoplasmic and nuclear maturation	Vitale et al., 2022 [55]
ROS–miRNA interaction	miR-21, miR-155 ↑ via NF-κB	GCs	Indirect: anti-apoptotic GC signaling → oocyte survival	Follicular integrity, luteinization	Andronico et al., 2023 [56]
Metabolic synchronization	ROS → xCT (SLC7A11), MCTs, glycolytic enzymes	CCs–Oocyte	Lactate & pyruvate transport, GSH synthesis precursors	Redox buffering, energy supply	Artini et al., 2022 [24]
Circadian redox integration	BMAL1–CLOCK, redox loops, NAD+/ROS feedback	GCs, Oocyte	Temporal alignment of redox & meiotic competence	Fertilization timing, ovarian aging prevention	Sato and Greco 2021 [57]

This table compiles selected mechanistic studies demonstrating how precisely regulated levels of ROS influence critical molecular pathways, cellular targets, and oocyte-specific outcomes during follicular development and ovulation. The referenced literature was chosen to present established and contemporary findings that illustrate various methodological approaches (e.g., human, animal, in vitro models), thereby providing a comprehensive overview of the physiological role of ROS in the ovarian environment.

## 3. Oocyte Maturation and OS: When Redox Balance Fails

### 3.1. OS and Its Cellular Triggers in the Ovarian Follicle

While in ovarian folliculogenesis ROS are necessary for physiological signaling, the change from redox homeostasis to OS has negative effects on oocyte quality, fertilizing capacity, and early embryonic development [58]. An overabundance of ROS—that is, superoxide (O_2_^−^), hydrogen peroxide (H_2_O_2_), and hydroxyl radicals (^•^OH)—that overwhelms antioxidant defense systems including glutathione (GSH), superoxide dimutase (SOD), catalase, and peroxiredoxins defines OS [59]. This imbalance in the ovarian milieu could be brought on by intrinsic elements such age, PCOS, and endometriosis or by extrinsic causes including smoking, environmental pollutants, or inadequate ovarian stimulation techniques used in in vitro fertilization (IVF).

### 3.2. Mitochondrial Dysfunction and Bioenergetic Collapse During Oocyte Maturation

The mitochondrion serves as a primary source and pertinent target of ROS, rendering it a significant focal point of OS. Mitochondria are necessary not only for the synthesis of adenosine triphosphate (ATP) but also for calcium buffering, redox management, and the regulation of apoptotic pathways inside the oocyte. Mature metaphase II oocytes possess a particularly high quantity of these organelles, which are distributed throughout the ooplasm [60]. Consequently, they are susceptible to OS due to diminished capacity for mitophagy and DNA repair mechanisms.

Excessive ROS production leads to the irreversible opening of the mitochondrial permeability transition pore (mPTP), resulting in matrix enlargement, cristae degradation, and the collapse of the mitochondrial membrane potential (ΔΨm) [61]. Oxidative phosphorylation relies on the electrochemical gradient; thus, its absence diminishes ATP production. During oocyte maturation, ATP is crucial for microtubule polymerization, actin cytoskeleton remodeling, chromosomal congression, and spindle orientation [62]. Decreased ATP production disrupts these processes, hence impacting spindle organization and kinetochore attachment, increasing the likelihood of aneuploidy and meiotic non-disjunction.

### 3.3. Genomic Instability and Epigenetic Vulnerability Induced by ROS

ROS simultaneously harm mitochondrial DNA (mtDNA), which is devoid of protective histones and insufficiently supplied with nucleotide excision and base excision repair enzymes. Oxidative lesions, particularly point mutations and deletions, frequently arise in genes encoding components of the ETC, especially in complexes I and III [63]. Dysfunctional electron transport exacerbates ROS leakage, hence perpetuating a deleterious feedback loop. OS alters the expression or activity of fusion–fission proteins, such as mitofusin-1/2 (MFN1/2), optic atrophy protein 1 (OPA1), and dynamin-related protein 1 (DRP1), thereby disrupting mitochondrial dynamics and leading to abnormal mitochondrial morphology and misplacement within the oocyte cytoplasm [64]. This diminishes the spatial availability of ATP in critical areas and inhibits proper mitochondrial aggregation around the meiotic spindle.

The release of cytochrome c from the mitochondrial intermembrane space can lead to excessive ROS, which may trigger intrinsic death signals. This association between procaspase-9 and apoptosis protease-activating factor 1 (APAF1) facilitates the formation of the apoptosome, hence activating executioner caspases such as caspase-3 and caspase-7 [65]. Even following fertilization, this pathway induces programmed cell death of the egg or sublethal damage that undermines its developmental competence. In addition to mitochondrial damage, OS significantly influences meiotic growth and the cell cycle. ROS modulate cyclin-dependent kinase (CDK) activity under normal physiological conditions by reversibly oxidizing cysteine residues on regulatory subunits, hence maximizing oocyte development [66]. CDK1, often referred to as cell division cycle 2 kinase (CDC2), undergoes oxidative modification when ROS exceed the neutralizing capacity of endogenous antioxidants, resulting in functional impairment. CDK1, in conjunction with cyclin B1, constitutes an essential element of the maturation-promoting factor (MPF); thus, its inactivation halts meiotic progression [67].

Cell division cycle phosphatase 25B (CDC25B) is a crucial target as it eliminates inhibitory phosphates from CDK1, hence activating MPF. OS diminishes CDC25B nuclear localization and activity, hence preventing the initiation of germinal vesicle disintegration and causing the egg to remain arrested at the prophase I stage. In oxidative conditions, MAPK pathways, including extracellular signal-regulated kinase 1/2 (ERK1/2) and MOS kinase, which are essential for cytoplasmic polyadenylation and the translation of critical mRNAs, are concurrently downregulated [68].

In addition to undermining meiotic regulation and bioenergetic potential, OS inflicts considerable genotoxic harm that directly jeopardizes oocyte genomic and developmental integrity. The oxidation of nucleotide bases, especially guanine, is one of the most significant chemical damages inflicted by ROS [69]. The distinctive lesion that can mispair with adenine during DNA replication is the oxidation product 8-oxo-2′-deoxyguanosine (8-oxo-dG). Despite the egg being a terminally differentiated cell that does not undergo division prior to fertilization, the persistence of such lesions suggests the potential for mutational transfer to the embryo, thereby affecting early cleavage-stage integrity and possibly impairing blastocyst development [70].

The inherently diminished DNA repair capability of the oocyte heightens its vulnerability to oxidative DNA damage. Transcriptional activity in the oocyte is predominantly inhibited during maturation, with repair mechanisms restricted to preloaded maternal transcripts and proteins. This limitation significantly hinders homologous recombination, essential for addressing mild oxidative damage and base excision repair (BER). During the transition from the germinal vesicle to metaphase II, critical enzymes—such as 8-oxoguanine DNA glycosylase (OGG1), poly (ADP-ribose) polymerase 1 (PARP1), and DNA polymerase β—exhibit diminished quantities or reduced activity [71,72]. Consequently, oxidative damage accumulates in both coding and regulatory regions of the genome, affecting gene expression as well as chromatin organization.

A robust DDR is activated when oxidative damages exceed the oocyte’s repair capacity. The phosphorylation of the histone variation H2AX is mediated by ataxia telangiectasia mutated (ATM) and ataxia telangiectasia and Rad3-related (ATR) kinases, leading to the formation of γH2AX foci at sites of injury [73]. This initiates a checkpoint cascade in which checkpoint kinase 2 (CHK2) halts the cell cycle and forms repair complexes. If damage remains unresolved, the tumor suppressor p53 is stabilized and transcriptionally produces pro-apoptotic proteins such as BCL2-associated X protein (BAX), BCL2-interacting mediator of cell death (BIM), and phorbol-12-myristate-13-acetate-induced protein 1 (PMAIP1/NOXA) [74,75]. These components converge on the mitochondrial outer membrane to release cytochrome c and enhance apoptotic signals.

The ramifications of ROS-induced DNA damage extend beyond mere structural genomic instability. For proper zygotic genome activation (ZGA), the oocyte must experience epigenetic reprogramming, which encompasses global DNA demethylation and chromatin remodeling throughout early development [76]. By disrupting enzymes involved in active DNA demethylation, particularly the ten-eleven translocation (TET) family of dioxygenases, ROS perturb this meticulously regulated epigenetic landscape. These enzymes require Fe2^+^ and α-ketoglutarate as cofactors, both of which are susceptible to oxidative inactivation, resulting in inadequate hydroxylation of 5-methylcytosine or insufficient promoter demethylation [77].

This could result in inappropriate gene suppression or activation, dysregulated transcriptional patterns, and loss of developmental competence.

Moreover, OS contributes to telomere attrition, a significant factor affecting oocyte quality. Telomeric DNA, abundant in guanine repeats (TTAGGG), is highly susceptible to oxidation. Meiosis compromises chromosomal stability by oxidative telomere shortening and reduced synthesis of telomerase reverse transcriptase (TERT). Telomere dysfunction in aged or metabolically stressed oocytes correlates with spindle disorganization, erroneous chromatid segregation, and a significant increase in embryonic aneuploidy. Exogenous antioxidants, including melatonin and resveratrol, have demonstrated in animal experiments the potential to reduce ROS levels, upregulate TERT expression, and enhance the stability of the shelterin complex, thereby preserving telomere length.

### 3.4. Redox Regulation by Non-Coding RNAs: microRNAs and lncRNAs

The impact of OS on non-coding RNA networks has emerged as a crucial mechanism linking cellular redox status to oocyte quality. Both miRNAs and lncRNAs participate in post-transcriptional and epigenetic regulation throughout the cumulus–oocyte axis, hence affecting follicular responses to environmental and metabolic stimuli [78]. These regulatory RNAs are variably synthesized during OS, hence affecting critical genes associated with mitochondrial dynamics, meiotic development, apoptosis, and antioxidant defense. ROS exposure directly modulates the expression of specific microRNAs, hence altering the levels of antioxidant enzymes and transcripts associated with apoptosis. Under oxidative conditions, the overexpression of miR-21 in GCs and induces temporary anti-apoptotic responses via PDCD4 and PTEN, hence enhancing short-term survival. Prolonged elevation of miR-21 can inhibit necessary apoptotic clearance, resulting in abnormal follicular structure and diminished oocyte maturation [79].

Conversely, OS diminishes the expression of miR-132, miR-212, and miR-399, which are associated with cytoplasmic maturation and oocyte–granulosa communication. miR-378 regulates the production of PGC1α and SIRT1, which are primary regulators of mitochondrial biogenesis, whereas miR-132 and miR-212 regulate EGF-like growth factors [80]. The loss of these miRNAs impairs mitochondrial function, disrupts metabolic support to the oocyte, and leads to cytoplasmic deficiency. A significant outcome of ROS is the elevation of microRNAs targeting crucial antioxidant genes. Follicles under OS significantly overexpress miR-23a and miR-141. MiR-141 inhibits SOD2, resulting in the accumulation of superoxide radicals in mitochondria, whereas miR-23a decreases GPX3, hence reducing extracellular peroxidase activity [81]. These alterations sustain redox imbalance and create a feedback loop of oxidative damage involving miRNA deregulation.

Through chromatin remodeling, transcriptional control, and ceRNA processes, lncRNAs simultaneously act as upstream regulators of gene expression and miRNA activity. The redox-sensitive physiology of follicles mostly centers on H19. The downregulation under OS corresponds with reduced IGF1 expression, impaired PI3K–AKT signaling, and disrupted cumulus expansion [82]. Furthermore, H19 functions as a miRNA sponge, particularly for the let-7 family, and its suppression leads to excessive let-7 activity, hence inhibiting targets like as HMGA2 and IGF1R, both essential for oocyte competency [83]. MALAT1, an important lncRNA involved in alternative splicing and nuclear retention of redox-responsive transcripts, has its expression diminished by ROS in CCs and GCs, thereby impairing the splicing of antioxidant defense genes and disrupting the transcripts required for GSH synthesis and calcium homeostasis [84]. Furthermore, the silencing of MALAT1 affects ATP production and mitochondrial membrane potential, hence indirectly facilitating inadequate cytoplasmic maturation. Multilayered regulatory networks, established through interactions between lncRNAs and miRNAs, respond dynamically to oxidative cues. Animal models have demonstrated that the H19-let-7-GSK3β and MALAT1-miR-200c-NRF2 pathways associate non-coding RNA dysregulation with spindle abnormalities, chromatin condensation inadequacies, and suboptimal fertilization results.

### 3.5. Antioxidant Systems and Clinical Relevance in Assisted Reproduction

The efficacy of the oocyte’s intrinsic antioxidant systems largely dictates its ability to endure OS. In conjunction with non-enzymatic molecules such as GSH, enzymatic antioxidants like SOD, CAT, and GPX, under equilibrated redox conditions, mitigate ROS and restrict oxidative damage to lipids, proteins, and nucleic acids, thus preserving cellular homeostasis. Excessive ROS production—due to ageing, inflammation, metabolic disorders, or external stressors—rapidly surpasses these defenses, resulting in the functional depletion of the antioxidant system.

GSH is synthesized in abundance within CCs and transported to the oocyte through gap junctions in the follicular environment; it also serves as a principal regulator of redox buffering. GSH is rapidly oxidized to GSSG during OS, hence diminishing the GSH/GSSG ratio and impairing the oocyte’s capacity to detoxify H_2_O_2_ and lipid peroxides [85]. Simultaneously, the activity of SOD and CAT diminishes, typically due to the depletion of metal cofactors and the oxidative inactivation of cysteine-rich catalytic domains, hence exacerbating the accumulation of superoxide and hydroxyl radicals [86].

In pro-oxidant situations, especially in FF from women with endometriosis, PCOS, or advanced reproductive age, GPX, which reduces H_2_O_2_ utilizing GSH as a substrate, is likewise downregulated.

Increasing signs of oxidative damage distinctly demonstrate the biological impact of antioxidant deficiency. Elevated levels of MDA, indicative of polyunsaturated lipid peroxidation, are frequently observed in FF from patients exhibiting poor ovarian response or failure in IVF cycles [87]. Comparable high levels of 8-oxo-dG indicate oxidative modification of guanine residues in both nuclear and mitochondrial DNA, hence jeopardizing genomic integrity and transcriptional accuracy during oocyte maturation. Carbonylated proteins and nitrated tyrosine residues in FF indicate oxidative modifications of structural and enzymatic proteins, potentially affecting spindle organization, organelle distribution, and cytoskeletal remodeling [88]. Clinical investigations have shown that a reduced fraction of recovered MII oocytes, diminished fertilization rates, impaired cleavage stage development, and decreased blastocyst formation are strongly connected with lower levels of GSH, SOD, and GPX in FF [89]. Moreover, patients undergoing IVF with elevated MDA levels in FF frequently have diminished implantation potential and inferior embryo fragmentation scores. Persistent redox imbalance has been observed in women experiencing unexplained infertility or repeated implantation failure, suggesting that subclinical OS may underlie otherwise inexplicable reproductive anomalies.

Numerous interventional studies have examined whether antioxidant supplementation might mitigate oxidative depletion and improve fertility. Numerous reports indicate that oral CoQ10, NAC, resveratrol, or melatonin elevate GSH levels, rejuvenate mitochondrial function, and reduce MDA in FF. In women with diminished ovarian reserve or advanced maternal age, these changes are typically accompanied by an increased quantity of high-quality MII oocytes, elevated fertilization rates, and improved clinical pregnancy and live birth rates [90]. The interplay between oocyte quality and antioxidant defense capacity appears to be contingent upon both dosage and duration. Excessive supplementing highlights the necessity for tailored redox regulation in IVF procedures, as it may disrupt the natural ROS signals essential for follicular rupture and luteinization. In FF, the assessment of redox markers such as GSH, SOD, MDA, and 8-oxo-dG is becoming recognized as a valuable diagnostic and prognostic method in ART.

Table 2 enumerates redox-sensitive signaling pathways and enzymes (e.g., Nrf2, MAPK, aldose reductase) alongside both traditional and novel OS-related biomarkers (e.g., GSH, SOD, GPX, MDA, 8-oxo-dG). It elucidates their physiological functions within the follicular milieu, the molecular consequences of their dysregulation in oxidative circumstances, and their reported associations with oocyte maturation, fertilization, embryo quality, and IVF success. The featured publications encompass several clinical situations, such as PCOS, endometriosis, and ovarian ageing, thereby establishing a translational connection between biological causes and therapeutic applications.

**Table 2:** Overview of OS-related biomarkers and pathways in oocyte quality and IVF outcomes.

Marker	Role in Follicular Environment	Effect of OS	Clinical Correlation in IVF	Key References
GSH	Major intracellular antioxidant; detoxifies H_2_O_2_ and lipid peroxides	Depleted under stress; reduced GSH/GSSG ratio	Low levels linked to lower MII yield and fertilization rates	Neyroud et al., 2022 [91]
SOD	Converts superoxide radicals into H_2_O_2_	Inactivated by ROS; impaired superoxide clearance	Low activity correlates with embryo fragmentation	Muraoka et al., 2020 [92]
CAT	Breaks down H_2_O_2_ into water and oxygen	Reduced activity; diminished H_2_O_2_ detoxification	Decreased levels found in poor responders	Neyroud et al., 2022 [91]
GPX	Reduces H_2_O_2_ using GSH as substrate	Downregulated expression; decreased peroxide reduction	Correlated with embryo quality and pregnancy rates	Andronico et al., 2021; Meseguer et al., 2006 [56,93]
MDA	End-product of lipid peroxidation; indicates OS	Increased levels; associated with poor oocyte and embryo quality	High levels associated with failed IVF and poor implantation	Muraoka et al., 2020; Yalcinkaya et al., 2023 [92,94]
8-oxo-dG	Marker of oxidative DNA damage	Elevated in FF; linked to DNA fragmentation	Associated with advanced age and poor IVF outcomes	Nori and Helmi 2023 [95]
CoQ10	Supports mitochondrial function and ATP production	Restores mitochondrial redox balance; reduces oxidative damage	Improves oocyte morphology and pregnancy outcomes	Neyroud et al., 2022 [91]
NAC	Precursor for GSH synthesis; scavenges free radicals	Enhances GSH levels; reduces lipid peroxidation	Beneficial in women with DOR and OS	Tenorio et al., 2021 [96]
Melatonin	Mitochondrial-targeted antioxidant; improves oocyte competence	Increases MII oocyte yield and embryo quality	Improves fertilization and clinical pregnancy rates	Song et al., 2016 [97]
GPX4, SOD1/2, CAT, MDA	Antioxidant enzymes and lipid peroxidation regulators	Enhanced with glycine supplementation; reduced lipid peroxidation	Improved cleavage and blastocyst rate in porcine oocytes (model)	Gao et al., 2023 [98]
Nrf2, Sirt, MAPK, AKT, FoxO	Molecular pathways involved in OS response and ovarian aging	Pathway dysregulation promotes mitochondrial damage, apoptosis, aging	Potential for antioxidant therapies to delay ovarian aging	Yan et al., 2022 [99]
General ROS, light-induced OS	Endogenous and exogenous ROS generation during ART	Photooxidation exacerbates ROS levels during ART handling	Recommendations for antioxidant strategies in ART	Mauchart et al., 2023 [100]
SOD, GPX, 8-oxo-dG, MDA, p16, p21	Antioxidant and senescence-related markers in FF of endometriosis patients	Rapamycin reduced OS markers, senescence, improved FF profile	Improved fertilization, implantation, and live birth rates with rapamycin	Fan et al., 2023 [101]
Aldose reductase, ROS	Polyol pathway regulation and OS in ovarian cells	Hyperandrogenism induces polyol pathway → ROS ↑ → follicular dysfunction	Explains oxidative mechanism in PCOS; potential therapeutic target	Wang et al., 2022 [102]

Thorough review of OS biomarkers, antioxidant enzymes, redox-regulated pathways with their molecular functions, reactions to OS, clinical correlations in IVF, and important supporting research released between 2020 and 2024.

## 4. OS and Embryonic Development

The early embryo, mostly reliant on maternally inherited transcripts and organelles, has rapid cleavage divisions and significant epigenetic reprogramming events post fertilization. Preimplantation embryos exhibit heightened sensitivity to OS during this transitional phase, characterized by metabolic immaturity and genomic silence before zygotic genome activation. In this phase, accumulated ROS impair subcellular structures, disrupt redox-sensitive signaling systems, and ultimately diminish developmental competence.

Due to the absence of protective histones and inadequate repair mechanisms, mtDNA is a primary molecular target of ROS in the early embryo and exhibits considerable sensitivity. Mitochondria, being maternally inherited and exhibiting limited proliferation during cleavage stages, serve as a stationary reservoir of oxidative damage. ROS-induced lesions, such as 8-oxo-dG, impair the transcription of genes encoding ETC complexes I and III, resulting in inefficient electron transfer and excessive ROS leakage, so creating a self-perpetuating cycle of mitochondrial dysfunction. The loss of ΔΨm due to this damage hinders ATP synthesis and activates the mPTP, consequently releasing cytochrome c into the cytoplasm. Cytochrome c binding to APAF1 initiates apoptosome formation and the recruitment and activation of caspase-9. Upon activation of downstream caspases (e.g., caspase-3, -7), the integrity of the blastocyst is compromised, resulting in a reduced total cell count and subsequent blastomere apoptosis. Additionally, the embryo’s capacity for metabolic flexibility is further constrained by ROS-induced mitochondrial damage, which diminishes the expression of crucial regulators of mitochondrial biogenesis such as PGC-1α and NRFs. This energy-deficient state is crucial for proper embryo cleavage and compaction, as it undermines spindle assembly, epigenetic remodeling, and ion homeostasis. In IVF scenarios, where in vitro treatment may elevate ROS levels, such mitochondrial anomalies directly influence developmental competence, implantation potential, and clinical outcomes.

In addition to causing mitochondrial damage, ROS profoundly alter the epigenetic landscape of the early embryo. ZGA and lineage specification are fundamentally reliant on global DNA demethylation and the reestablishment of histone modifications during preimplantation development. Active DNA demethylation, involving the conversion of 5-mC to 5-hmC, is contingent upon TET enzyme activity, which is disrupted by elevated levels of ROS. This deficiency leads to abnormal methylation retention in promoter regions of developmentally regulated genes, resulting in transcriptional dysregulation and asynchronous cleavage. Under oxidative conditions, redox-sensitive chromatin remodelers, like as HDACs and HMTs, are concurrently inhibited, resulting in histone hyperacetylation, chromatin decondensation, and altered nucleosome orientation. Chromatin abnormalities compromise the accuracy of transcriptional reprogramming and disrupt transcription factor binding, hence diminishing blastocyst viability and implantation potential. Simultaneously, oxidative damage occurs to cytoskeletal components essential for first cleavage divisions. During mitosis, ROS-induced oxidation and carbonylation of α-tubulin and actin filaments distort spindle morphology, kinetochore attachment, and chromosomal alignment. Blastomeres have irregular cleavage planes, increased aneuploidy, and heightened fragmentation—characteristics indicative of suboptimal embryos often observed in IVF contexts.

Table 3 presents a synthesized overview of the molecular pathways by which ROS adversely affect early embryonic development. The data emphasizes critical systems such as spindle integrity, ZGA, epigenetic regulation (DNA methylation and histone modifications), and mitochondrial function. Energy failure arises from mitochondrial impairment, characterized by mtDNA degradation, dissipation of ΔΨm, and the opening of the mPTP, which triggers apoptotic cascades through cytochrome c and APAF1. ROS concurrently disrupt TET and HDAC enzymes, hence impairing the epigenetic remodeling essential for gene activation and chromatin architecture. Oxidation of tubulin and actin leads to cytoskeletal disintegration, which impedes mitotic advancement, hence enhancing fragmentation and aneuploidy. Highlighting the necessity of redox homeostasis for embryonic viability in IVF contexts, these redox-induced disruptions together lead to inadequate cleavage dynamics, blastocyst stagnation, and diminished implantation potential.

## 5. Antioxidant Approaches to Alleviate OS in In Vitro Fertilization

Our primary objective for this review was to provide a mechanistic synthesis; nevertheless, we recognize that incorporating quantitative data is essential for enhancing its clinical relevance. In this instance, it is crucial to note that typical concentrations of reactive oxygen species (ROS) in follicular fluid, such as hydrogen peroxide (H_2_O_2_), generally range from 25 to 50 µmol/L equivalents. These levels facilitate the maturation and ovulation of the egg. Concentrations exceeding 100 µmol/L have been associated with oxidative damage, DNA fragmentation, and a reduced likelihood of fertilization. Similarly, the antioxidant therapies discussed in this review have been utilized at various degrees in distinct clinical and experimental contexts. Administering 3–6 mg of melatonin orally each day for 30–60 days prior to IVF has been shown to enhance egg quality and embryo development. Individuals have consumed coenzyme Q10 (CoQ10) in dosages ranging from 200 to 600 mg daily for a duration of 8 to 12 weeks to enhance mitochondrial function and improve oocyte competence. Women with diminished ovarian reserve been administered 600 to 1800 mg of N-acetylcysteine (NAC) daily. In vitro maturation medium containing glycine concentrations ranging from 2.5 to 5 mM has demonstrated antioxidant effects in animal models. Rapamycin was administered to IVF patients with endometriosis as a brief pre-treatment to mitigate oxidative and age-related damage at a dosage of 1–2 mg/day. Recent investigations yield figures that establish a scientific foundation for the potential application of redox-modulating pharmaceuticals in reproductive medicine.

Due to the various detrimental effects of OS on oocyte quality, embryonic growth, and implantation potential, specific antioxidant therapies have become a prospective complement in ART. These techniques seek to re-establish redox equilibrium, safeguard mitochondrial integrity, and maintain the molecular fidelity of early development.

Within the context of ART, numerous studies have evaluated endogenous antioxidant regulation and exogenous antioxidant supplementation as strategies to mitigate ROS accumulation [103].

The thiol-dependent antioxidant system, comprising GSH, SOD, CAT, and GPX, functions synergistically to detoxify superoxide anions, H_2_O_2_, and lipid hydroperoxides, thereby serving as the primary line of cellular defense [59]. As a direct scavenger of hydroxyl radicals, GSH also serves as a substrate for GPX-facilitated reduction of peroxides. However, in vitro conditions during ART—particularly elevated O_2_ concentrations, absence of FF antioxidants, photo-oxidative exposure from ambient light, and repetitive pipetting—induce supraphysiological ROS levels that can overwhelm and diminish these defensive mechanisms.

This perturbation at the molecular level impacts the redox-sensitive regulation of transcription factors, including NRF2, a principal regulator of antioxidant response elements (AREs). Under typical conditions, NRF2 is sequestered in the cytoplasm by KEAP1; however, OS induces conformational alterations in KEAP1, facilitating the release of NRF2 to translocate into the nucleus [104]. Upon entry, NRF2 regulates GCLC, GCLM (for GSH synthesis), GPX4, SOD1, and CAT, among other antioxidant genes. Excessive or sustained ROS generation, prevalent in in vitro culture conditions, might diminish NRF2 signaling through the oxidation of nuclear import proteins or activation of proteasomal degradation pathways, consequently reducing endogenous antioxidant gene expression [105].

The NADPH pool necessary for GSH replenishment by glutathione reductase, as well as ATP generation, is further compromised by mitochondrial OS. The redox imbalance elevates lipid peroxidation, resulting in the accumulation of MDA and 4-HNE, which can form protein adducts and inactivate crucial metabolic enzymes. Gao et al. (2023) demonstrated that the overexpression of SOD2, GPX4, CAT, and PGC-1α, along with glycine supplementation—a precursor of GSH—enhanced mitochondrial redox state, decreased MDA levels, and improved mitochondrial function. These effects diminished ferroptosis, a lipid ROS-induced variant of programmed cell death detrimental to oocyte maturation and embryonic development, and reinforced mitochondrial membrane integrity [98].

The oxidative inactivation of SOD and CAT enzymes in granulosa and cumulus cells disrupts the follicular environment, leading to premature luteinization and diminished paracrine support for the egg. Mauchart et al. (2023) emphasized that photo-oxidation, sometimes overlooked in IVF circumstances, can rapidly generate singlet oxygen and hydroxyl radicals, hence straining antioxidant mechanisms. This underscores the urgent need to optimize cultural settings, particularly the management of air oxygen and the minimization of light exposure. The artificial oxidative environment of ART treatments creates a stress threshold that exceeds natural capability, notwithstanding the robustness of the endogenous antioxidant network under physiological conditions. This justifies intentional exogenous antioxidant therapies aimed at restoring redox equilibrium, enhancing mitochondrial metabolism, and improving reproductive outcomes [100].

An extended imbalance between ROS production and antioxidant defense mechanisms activates molecular pathways associated with inflammation, hence undermining follicular integrity.

ROS in the ovarian environment activate NF-κB, a redox-sensitive transcription factor, thereby regulating the expression of pro-inflammatory cytokines such as TNF-α, IL-1β, and IL-6. These mediators not only augment OS but also disrupt glucocorticoid metabolism and survival by propagating a localized inflammatory response [106]. Mechanistically, sustained NF-κB activation alongside simultaneous stimulation of the JNK and p38 MAPK pathways induces mitochondrial dysfunction by ΔΨm collapse, cytochrome c release, and activation of APAF1–caspase-9. Increased levels of TNF-α and IL-1β concurrently enhance NOX-derived ROS production and upregulate iNOS, resulting in excessive synthesis of peroxynitrite and nitration of critical mitochondrial proteins [107]. This induces irreversible oxidative damage to mtDNA and diminishes the efficacy of the ETC complex, hence exacerbating ATP depletion and meiotic irregularities.

Moreover, inflammatory cytokines inhibit NRF2 activity and its downstream antioxidant response elements, such as HO-1, GCL, and GPX. The consequent decrease in intracellular GSH undermines redox buffering in both GCs and oocytes, hence heightening their vulnerability to ferroptosis and apoptotic degeneration [108]. These molecular changes restrict cytoplasmic maturation, disrupt the spindle apparatus, and diminish the ability of the COC to respond to gonadotropin signaling. Increased levels of IL-6 and TNF-α in FF are associated, based on clinical data from IVF cohorts, with a diminished percentage of mature MII oocytes, elevated rates of embryo fragmentation, and suboptimal blastulation [109]. These findings suggest that through closely interconnected redox-inflammatory pathways, inflammatory mediators serve not only as markers of OS but also actively compromise oocyte competence and embryonic development. Targeting this axis therapeutically may enhance ART success and improve follicular resilience.

Melatonin, an indoleamine with amphiphilic properties that can permeate cellular and mitochondrial membranes, is among the most extensively studied antioxidant therapies in ART. Melatonin functions as an indirect enhancer of natural antioxidant defenses and a direct ROS scavenger during oocyte maturation and embryo development [110]. Melatonin protects mitochondrial integrity by stabilizing ΔΨm, preventing mPTP opening, and inhibiting cytochrome c release into the cytosol, hence obstructing the activation of the APAF1–caspase-9 apoptotic pathway. These actions facilitate meiotic spindle assembly and chromosomal alignment while also supporting ATP synthesis [111]. Melatonin, at the molecular level, enhances the transcription of crucial antioxidant enzymes such as SOD, CAT, and GPX by activating the NRF2–ARE signaling pathway. By upregulating GCLC and GCLM, it enhances GSH synthesis, hence restoring the GSH/GSSG ratio essential for redox equilibrium during cytoplasmic and nuclear development [112].

Research conducted by Muraoka et al. (2020) demonstrated that melatonin significantly enhances MII oocyte production and blastocyst development while reducing levels of 8-oxo-dG and MDA in both ocells and embryos. This was followed by increased SOD and GPX activity, which corroborates its role in enhancing mitochondrial antioxidant capacity. Furthermore, melatonin diminishes pro-apoptotic BAX and modifies gene expression associated with mitochondrial biogenesis and metabolic regulation, including PGC-1α, TFAM, and BCL2 [92]. Andronico et al. (2019) discovered that melatonin supplementation enhanced embryo quality and clinical pregnancy rates, especially in women with elevated oxidative stress, including poor responders and those of advanced maternal age [56]. Melatonin is a potent and therapeutically significant antioxidant in IVF operations, as it neutralizes ROS and upregulates redox-responsive gene networks. However, to enhance its application, further dose-response study and patient categorization are necessary.

N-acetylcysteine (NAC), serving as a precursor to glutathione (GSH), the predominant intracellular thiol antioxidant, is essential for enhancing the cellular redox environment. NAC facilitates the production of GSH by supplying cysteine, the rate-limiting amino acid in GSH synthesis, hence enhancing the enzymatic function of γ-glutamylcysteine synthetase. This enhances GPX activity, particularly GPX4, which is crucial for reducing lipid hydroperoxides [113]. NAC maintains the GSH/GSSG balance and reduces ROS levels, hence averting spindle disorganization and DNA fragmentation in oocytes subjected to oxidative stress. NAC has been shown to activate the NRF2 signaling pathway by modifying KEAP1 cysteine residues, hence stabilizing NRF2 and enhancing the transcription of genes such as SOD, CAT, GCLM, and GCLC [114]. In women with diminished ovarian reserve, whose innate antioxidant defenses are frequently compromised due to mitochondrial ageing or granulosa cell senescence, NAC therapy has been associated with improved egg maturation, decreased fragmentation, and enhanced blastocyst development in the setting of assisted reproductive technology [19].

The production of mitochondrial ATP through oxidative phosphorylation relies on coenzyme Q10 (CoQ10), a crucial electron carrier within ETC complexes I and III. CoQ10 facilitates the establishment of proton gradients across the inner mitochondrial membrane in oocytes, where metabolic demands are elevated during maturation and fertilization, hence enhancing ATP synthase activity [115]. Reduced (ubiquinol) and oxidized (ubiquinone) forms generate coenzyme Q10; its redox cycling directly neutralizes lipid-derived radicals and inhibits cardiolipin oxidation, a critical event in mitochondrial permeability transition pore (mPTP) opening and cytochrome c release [116]. CoQ10 inhibits the initiation of the APAF1–caspase-9 apoptotic cascade by stabilizing ΔΨm and preserving mitochondrial membrane integrity. CoQ10 enhances the transcription of nuclear-encoded mitochondrial genes and stimulates PGC-1α-mediated mitochondrial biogenesis, thereby augmenting both the quantity and quality of mitochondria [117]. In women exhibiting poor ovarian response or age-related mitochondrial decline, both clinical and experimental evidence suggest that CoQ10 supplementation can restore mitochondrial quantity and functionality in aged oocytes, hence enhancing MII rates, embryo quality, and pregnancy outcomes.

Glycine, a non-essential amino acid and structural element of GSH, exerts a complex influence on oocyte redox and metabolic regulation. According to Gao et al. (2023), glycine supplementation during in vitro maturation dramatically increased intracellular GSH and elevated redox-regulating genes such as GPX4, SOD1, SOD2, and CAT, which are essential for ROS detoxification. Through the stimulation of PGC-1α and PPARγ, glycine altered mitochondrial and lipid homeostasis, hence enhancing mitochondrial biogenesis and β-oxidation. Furthermore, glycine augmented the expression of SREBF1 and AMFR, which are implicated in lipid droplet formation and ER–mitochondria interactions, consequently bolstering peroxisomal antioxidant capacity [98]. The stabilization of membrane lipids and the reduction of MDA accumulation led to the unexpected inhibition of ferroptosis, a lipid ROS-dependent form of regulated necrosis. These alterations enhanced cytoplasmic competence and nuclear maturation, hence improving cleavage and blastocyst rates in vitro. In addition to its role in supporting antioxidants, glycine also influences oocyte transcriptional and metabolic remodeling.

Among women with endometriosis undergoing IVF, the therapeutic application of rapamycin, a particular mTOR inhibitor, has emerged as an effective strategy to address oxidative and senescence-related dysfunction in the follicular environment. Reduced levels of MDA and 8-hydroxy-2′-deoxyguanosine (8-OHdG) in FF indicate that short-term pre-treatment with rapamycin prior to ovarian stimulation greatly mitigated oxidative damage in the cohort research conducted by Fan et al. (2023) [101]. These signs indicate a reduction in DNA oxidative damage and lipid peroxidation, respectively.

Rapamycin diminishes, at the molecular level, significant senescence-associated markers p16^INK4a and p21^CIP1/WAF1, which are both downstream targets of p53 and crucial mediators of cell cycle arrest. Their inhibition indicates a decrease in senescence-induced dysfunction in granulosa and CCs, hence preserving the paracrine signaling essential for oocyte maturation [118]. Rapamycin enhances autophagic flow by inhibiting mTORC1, thereby facilitating the removal of faulty organelles that accumulate due to oxidative damage and promoting mitophagy of damaged mitochondria. The restoration of intracellular homeostasis is essential for diminished ROS production, enhanced energy metabolism, and protection against cell death [119].

Clinically treated patients with rapamycin had no documented side effects, reduced ovarian stimulation duration, increased numbers of retrieved and MII oocytes, and significantly enhanced rates of fertilization, implantation, clinical pregnancy, and live births. These findings underscore the dual role of rapamycin as an antioxidant and anti-senescence agent, particularly in oxidative microenvironments such ovaries afflicted by endometriosis.

Mauchart et al. (2023) examined the often overlooked influence of iatrogenic ROS, ART operations, particularly in vitro culture (IVC) of gametes and embryos, alongside pharmacological interventions. Particularly in the blue spectrum (~400–500 nm), light exposure induces photo-oxidation of culture media and intracellular constituents such as riboflavin and tryptophan, resulting in the production of singlet oxygen and the creation of free radicals. This external OS impacts mitochondrial function, disrupts embryonic redox equilibrium, and modifies blastocyst development and cleavage patterns [100]. Photo-protective methods, such as amber light filters, red LED illumination, and minimized exposure durations, have been shown to maintain oocyte and embryo quality to counteract this issue. Additionally, it has been suggested to neutralize ROS produced in culture by including antioxidants such as vitamins C and E, melatonin, or N-acetylcysteine into the culture media. This would also aid in the preservation of ΔΨm. These methods aim to simulate a more typical environment by enhancing the embryonic antioxidant defense system and reducing mitochondrial dysfunction, hence optimizing ART outcomes.

Table 4 compares the most studied antioxidant treatments in assisted reproductive technologies, including melatonin, NAC, CoQ10, glycine, rapamycin, and sun protection measures. Each method is characterized by its primary molecular targets, mechanisms of redox modulation, and recorded clinical effects, which encompass alterations in oocyte quality, fertilization rates, blastocyst development, and pregnancy outcomes. Recent field-based experimental and clinical research is synthesized into data.

**Table 4:** Antioxidant strategies in IVF: molecular targets, mechanisms, and clinical impact.

Intervention	Primary Molecular Targets	Mechanism of Action	Clinical Impact	Key References
Melatonin	ΔΨm, SOD, GPX, NRF2, APAF1–caspase-9 axis	Mitochondrial antioxidant; enhances enzyme expression and prevents apoptosis	↑ MII oocytes, embryo quality, pregnancy rates	Muraoka et al. 2024, Andronico et al. 2019 [56,92]
NAC	GSH synthesis, NRF2–ARE, GPX4, GSH/GSSG ratio	Boosts thiol levels and GSH-mediated redox buffering; enhances meiosis	↑ Oocyte maturation, ↓ embryo fragmentation (especially in DOR)	Gu et al. 2024, Andronico et al. 2019 [56,120]
CoQ10	ETC Complexes I & III, ΔΨm, PGC-1α, BCL2, ROS detox	Supports ETC efficiency, ATP production, and mitochondrial integrity	↑ Oocyte competence, pregnancy rates (especially in aging)	Neyroud et al. 2022 [91]
Glycine	GPX4, SOD1/2, CAT, PGC-1α, PPARγ, ferroptosis pathways	Enhances antioxidant gene expression, lipid metabolism, and membrane stability	↑ Cleavage & blastocyst rates in vitro	Gao et al. 2023 [98]
Rapamycin	mTORC1, p16/p21, 8-OHdG, autophagy, mitochondrial clearance	Reduces senescence markers, ROS damage, and enhances mitophagy	↑ Fertilization, implantation, live birth rates (in endometriosis)	Fan et al. 2024 [101]
Light Protection	ROS from photo-oxidation, culture media protection	Reduces iatrogenic ROS through light shielding and media supplementation	↑ Embryo viability, ↓ ROS-induced developmental arrest	Mauchart et al. 2023 [100]

Legend: ΔΨm—mitochondrial membrane potential; SOD—superoxide dismutase; GPX—glutathione peroxidase; NRF2—nuclear factor erythroid 2-related factor 2; APAF1—apoptotic protease-activating factor 1; NAC—N-acetylcysteine; GSH—glutathione; ETC—electron transport chain; BCL2—B-cell lymphoma 2; CAT—catalase; PPARγ—peroxisome proliferator-activated receptor gamma; mTORC1—mechanistic target of rapamycin complex 1; 8-OHdG—8-hydroxy-2′-deoxyguanosine.

## 6. Clinical Implications

OS significantly influences oocyte quality, embryo growth, and implantation potential in ART. The therapeutic significance is progressively acknowledged, influencing embryo culture enhancement and individualized therapy approaches. Redox biomarkers, including MDA, 8-oxo-dG, the GSH/GSSG ratio, and the enzyme activity of SOD, CAT, and GPX, function as non-invasive indicators of mitochondrial performance, DNA integrity, and lipid peroxidation. Deviant levels in FF or serum are associated with diminished MII retrieval, decreased fertilization rates, and inferior embryo grading. In patients with PCOS, DOR, and AMA, redox profiling may inform modifications to COS and antioxidant supplementation.

Targeted antioxidant treatments, such as MEL, NAC, CoQ10, and GLY, provide cytoprotective benefits by stabilizing ΔΨm, inhibiting the formation of ROS and MDA, and enhancing the production of NRF2, SOD, and GPX. MEL promotes MII yield and ATP production, NAC restores GSH levels and reduces apoptosis, CoQ10 improves ETC efficiency and mitochondrial biogenesis, while GLY regulates lipid metabolism and ferroptosis through PGC-1α and GPX4.

Exosomal miRNAs have innovative therapeutic potential through the regulation of intracellular oxidative processes. miR-21, miR-132, and miR-146a modulate the PI3K–AKT–mTOR, KEAP1–NRF2, and PGC-1α–TFAM pathways, thereby enhancing redox equilibrium, mitochondrial proliferation, and ΔΨm stability. The dysregulation of miRNA cargo in PCOS and DOR leads to increased MDA, activation of caspase-3, and compromised spindle formation. Engineered exosomes or miRNA-enriched medium demonstrate potential in improving oocyte competency and IVF success rates. Collectively, our findings endorse the incorporation of redox-based diagnostics and treatments into ART to enhance oocyte quality and reproductive results.

## 7. Limitations and Future Directions

Robust data indicates that OS contributes to oocyte maturation failure, mitochondrial dysfunction, and impaired IVF outcomes; nonetheless, significant limitations hinder the full translational potential of current understanding. A significant limitation is the dependence on non-human models—specifically murine and porcine systems—which, although beneficial for clarifying ΔΨm, ROS dynamics, and apoptotic activation (via APAF1–caspase-9), fail to accurately mimic the human follicular microenvironment, encompassing exosomal miRNA composition and immune–redox interactions.

The synthesis is hindered by methodological discrepancies among studies. Fluctuations in O_2_ tension, antioxidant availability, outcome metrics, and ROS measurement methodologies—such as DCFDA and MitoSOX—impede reproducibility. Standardized thresholds enhance clinical usefulness; biomarkers such as MDA, 8-oxo-dG, GSH/GSSG, and SOD activity exhibit significant variability in baseline values. Variability also stems from the timing of sampling—during ovarian stimulation, at oocyte retrieval, or in embryo culture. Interventions such as MEL, NAC, CoQ10, and GLY enhance ΔΨm preservation, regulate ROS, and promote embryo growth. The optimal dosage, timing, and duration remain uncertain. Excessive exposure to antioxidants may diminish normal ROS-dependent signaling pathways essential for spindle assembly, PI3K–AKT–motor, and LH–EGF–ERK cascades, hence inducing reductive stress.

Exosomal miRNAs (e.g., miR-21, miR-132, miR-146a) have ambiguous roles in ART contexts, despite their significant influence on redox equilibrium. Modified exosome cargo influences genes such as GPX4, CAT, SOD2, and TFAM in conditions like PCOS, DOR, or AMA, hence impacting COC dynamics. Challenges in vesicle isolation, stability, and targeted miRNA mimicry remain unresolved.

Future endeavors should prioritize meticulously constructed randomized controlled trials that stratify women based on redox status (e.g., elevated FF MDA or diminished GSH) and employ multi-omics approaches (proteomics, metabolomics, miRNome) to establish predictive biomarkers for MII yield, blastocyst development, and implantation. Innovative nanocarriers—such as mitochondria-targeted antioxidants or exosome-based delivery systems—may provide precise modulation of OS pathways. Customized redox rebalancing tailored to specific oxidative profiles may enhance IVF outcomes more effectively than conventional antioxidant therapy.

## 8. Conclusions

In ART, OS represents a critical molecular impediment to optimal oocyte maturation, fertilization, and embryonic development. Meiotic restart, GC expansion, and ovulatory activities rely on normal levels of ROS; nevertheless, excessive ROS leads to ΔΨm collapse, mtDNA damage, and apoptosis via APAF1–CASP9 pathways. These disruptions compromise cytoskeletal integrity, hinder kinetochore–spindle interactions, and result in meiotic errors and aneuploidy.

Elevated ROS levels in the embryo result in fragmentation, reduced blastomere count, and diminished implantation potential. Subpar clinical outcomes have frequently been linked to increased biomarkers such as MDA and 8-oxo-dG. ART circumstances, characterized by elevated O_2_ levels, sun exposure, and mechanical stress, frequently surpass natural defense mechanisms (GSH, SOD, CAT, GPX) and intensify ROS production. Antioxidative strategies encompass MEL, NAC, CoQ10, GLY, and rapamycin, which have empirical support from several trials for restoring redox balance. These pharmaceuticals reduce markers of lipid peroxidation and DNA damage, enhance MII yield, and stimulate ATP synthesis. The use of antioxidants into IVM and embryo culture media has demonstrated potential to enhance embryo quality and increase conception rates.

The redox regulatory network is further augmented by exosomal miRNAs, such as miR-21, miR-132, and miR-146a. Their payload influences apoptotic resistance, GPX4, SOD2, CAT, and mitochondrial biogenesis, specifically regarding antioxidant gene expression. In PCOS and DOR, dysregulated exosomal signaling is associated with elevated ROS, ferroptosis, and impaired COC integrity. Experimental interventions utilizing modified exosomes have demonstrated improved ΔΨm, accelerated cleavage rates, and enhanced blastocyst development. Redox biology offers a promising translational framework in reproductive medicine. Prioritization of the validation of OS biomarkers for patient stratification, the standardization of antioxidant supplementation techniques, and the clinical translation of exosome-based treatments should be crucial to future advancements. In redox-compromised infertility phenotypes, the use of redox-targeted techniques into ART may enhance fertilization outcomes, embryo development, and live birth rates.

## Figures and Tables

**Table 3:** Embryonic OS pathways and developmental consequences.

Affected System	Primary ROS Effect	Developmental Consequence
Mitochondrial Function	mtDNA damage, ΔΨm loss, mPTP opening	ATP depletion, reduced blastocyst cell number
DNA Methylation	Inhibition of TET, impaired 5-hmC generation	Persistent methylation, transcriptional errors
Histone Modification	Inactivation of HDACs, histone hyperacetylation	Chromatin decondensation, faulty gene regulation
Cytoskeleton	Oxidation of tubulin and actin, spindle disruption	Fragmentation, aneuploidy, mitotic arrest
Zygotic Genome Activation	Epigenetic dysregulation, asynchronous gene expression	Delayed or failed embryo cleavage
Cellular Apoptosis	Cytochrome c release, caspase-9 activation	Blastomere loss, developmental arrest

Summary of main cellular systems impacted by OS during preimplantation embryogenesis, including with related molecular lesions, disrupted pathways, and their effect on embryo survival in ART environments.
